# Increasing Employee Awareness of the Signs and Symptoms of Heart Attack and the Need to Use 911 in a State Health Department

**Published:** 2004-06-15

**Authors:** Todd S Harwell, Crystelle C Fogle, Carrie S Oser, Lynda L Blades, Steven D Helgerson, Dorothy Gohdes, Michael R Spence, Drew E Dawson

**Affiliations:** Montana Department of Public Health and Human Services; Montana Department of Public Health and Human Services, Helena, Mont; Montana Department of Public Health and Human Services, Helena, Mont; Montana Department of Public Health and Human Services, Helena, Mont; Montana Department of Public Health and Human Services, Helena, Mont; Montana Department of Public Health and Human Services, Helena, Mont; Montana Department of Public Health and Human Services, Helena, Mont; Montana Department of Public Health and Human Services, Helena, Mont

## Abstract

**Introduction:**

Early recognition of the signs and symptoms of a heart attack can lead to reduced morbidity and mortality.

**Methods:**

A workplace intervention was conducted among 523 Montana state health department employees in 2003 to increase awareness of the signs and symptoms of heart attack and the need to use 911. All employees received an *Act in Time to Heart Attack Signs* brochure and wallet card with their paychecks. *Act in Time* posters were placed in key workplace areas. A weekly e-mail message, including a contest entry opportunity addressing the signs and symptoms of heart attack, was sent to all employees. Baseline and follow-up telephone surveys were conducted to evaluate intervention effectiveness.

**Results:**

Awareness of heart attack signs and symptoms and the need to call 911 increased significantly among employees from baseline to follow-up: pain or discomfort in the jaw, neck, or back (awareness increased from 69% to 91%); feeling weak, light-headed, or faint (awareness increased from 79% to 89%); call 911 if someone is having a heart attack or stroke (awareness increased from 84% to 90%). Awareness of chest pain, pain or discomfort in the arms or shoulders, and shortness of breath were more than 90% at baseline and did not increase significantly at follow-up. At baseline, 69% of respondents correctly reported five or more of the signs and symptoms of heart attack; 89% reported correctly at follow-up.

**Conclusion:**

This low-cost workplace intervention increased awareness of the signs and symptoms of heart attack and the need to call 911.

## Introduction

Heart disease continues to be the leading cause of death in the United States in 2003, with more than 1 million Americans experiencing a new or recurrent acute myocardial infarction (AMI) (1). Timely coronary reperfusion (e.g., angioplasty, thrombolytic therapy) and arrhythmia control can reduce morbidity and mortality in persons experiencing AMI (2). Reducing the time from the initial occurrence of symptoms to hospital arrival can increase the likelihood that these therapies are used early in the course of AMI.

In the United States, the median delay time in patients hospitalized with AMI was 2.1 hours in 1997, and 32% of patients with AMI had delay times of more than four hours (3). Persons experiencing AMI may delay seeking care because of a number of factors, including inadequate knowledge of signs and symptoms, attribution of symptoms to other non-serious conditions, or other barriers such as fear, concerns about cost, and embarrassment about calling emergency medical services (4-11). Additionally, most people in the United States who experience AMI transport themselves to medical care instead of using emergency medical services (12). However, persons who believe that their symptoms are serious are less likely to delay and more likely to use emergency medical services. In addition, women may experience an array of symptoms that differ from symptoms experienced by men (13). This difference may delay recognition and acute care for AMI among women.

Increasing early awareness of the signs and symptoms of AMI and the need to use the 911 emergency telephone system can reduce delays in seeking treatment, thus reducing morbidity and mortality. This is particularly important for rural and frontier communities, where individuals must travel long distances to tertiary care facilities.

In 2003, the Montana Department of Public Health and Human Services (MT DPHHS) conducted an intervention within the state health department to increase employee awareness of the signs and symptoms of a heart attack and the need to use 911. This report assesses the effectiveness of the intervention.

## Methods

### Setting

The intervention was conducted within the three MT DPHHS workplaces located in Helena, Mont (site one, site two, and site three). There were 523 employees at these three sites; 70% were female; and the mean age was 47.7 years.

### Intervention

**Figure 1:**
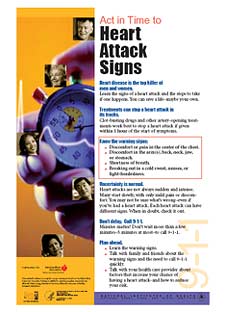
*Act in Time* poster developed by the National Institutes of Health to provide information on warning signs of heart attack and importance of calling 911 for emergency medical services.

Based on the original content of the Rapid Early Action for Coronary Treatment (REACT) research program developed by the National Institutes of Health (NIH), the *Act in Time* campaign provides information on the warning signs of heart attack and the importance of calling 911 for emergency medical services (http://www.nhlbi.nih.gov/ actintime/) (14). Our adaptation of *Act in Time* included several components designed specifically for a workplace intervention. First, all employees at the three sites received a one-time distribution of *Act in Time* brochures and wallet cards with their pay stubs (15). Second, *Act in Time* posters were placed on bulletin boards, in hallways, and in all bathrooms at each work site during the six-week test period (Figure 1). Third, also for six weeks, all employees received weekly e-mail messages and contest questions addressing the signs and symptoms of heart attack. Participants who answered the questions correctly were included in a weekly drawing for prizes (e.g., pedometers). Approximately one third of all employees participated in these weekly e-mail contests (participation ranged from 29% to 36%). The total estimated cost for intervention materials and staff time was $1037 (mean cost per employee = $1.98).

### Evaluation and data analysis

To evaluate this intervention, we conducted baseline and follow-up telephone surveys. The baseline survey took place over a two-week period in April 2003, and attempts were made to contact all 523 employees at each of the three sites. Respondents were asked a series of seven standardized questions about the signs and symptoms of heart attack from the Behavioral Risk Factor Surveillance System survey (Table 1) (16). The follow-up telephone survey was administered in July 2003 to the 401 employees responding to the initial baseline survey. The participants again were asked questions about the signs and symptoms of heart attack and the need to use 911. Respondents were also asked the following questions to assess their awareness of intervention activities:

- "In the past two months, have you seen posters in your workplace with information on heart attack signs and symptoms?" Those who responded yes were asked, "Where did you see these posters? Was it in the: hallways, stairwells, elevators, bathrooms?"
- "Did you participate in any of the weekly e-mail quiz contests on heart attack signs and symptoms?"
- "In the past two months, did you receive a brochure and wallet card on heart attack signs and symptoms with your paycheck?"

Data analyses were completed using SPSS version 10.0 statistical analysis software (Chicago, Ill). Independent t-tests and Pearson chi-square tests were used to compare the characteristics of respondents to the baseline and follow-up surveys. Pearson chi-square tests were used to assess differences in the proportion of respondents correctly identifying each individual sign and symptom of heart attack and the proportion identifying five or more signs and symptoms of heart attack at baseline compared to follow-up. Pearson chi-square tests were also used to assess the differences in the proportion of respondents correctly answering the question regarding the need to use 911 at baseline compared to follow-up.

## Results

Of the 523 employees, 401 (77%) completed the baseline survey. Of these 401 respondents, 337 (84%) completed the follow-up survey. There were no statistically significant differences in age between baseline and follow-up: baseline respondent mean age was 46.4 years (SD 9.3); follow-up respondent mean age was 46.6 years (SD 8.9). Nor were there statistically significantly differences in sex between baseline and follow-up: 71% of baseline respondents were women, and 72% of follow-up respondents were women (data not shown). Similarly, there were no differences in the proportion of respondents from each of the three work sites at baseline or follow-up (site one, 58% responded at baseline, 57% at follow-up; site two, 38% at baseline, 40% at follow-up; site three, 4% at baseline, 4% at follow-up).

Awareness of selected signs and symptoms increased significantly among employees from baseline to follow-up: pain or discomfort in the jaw, neck, or back (awareness increased from 69% to 91%); and feeling weak, light-headed, or faint (awareness increased from 79% to 89%) (Table 2). Awareness of chest pain, pain or discomfort in the arms or shoulders, and shortness of breath was greater than 90% at baseline and did not increase significantly at follow-up. The proportion of respondents who correctly reported that "sudden trouble seeing in one or both eyes" was not a sign or symptom of heart attack did not change significantly from baseline to follow-up. At baseline, 69% of respondents reported five or more of the signs and symptoms of heart attack correctly, and this increased to 89% at follow-up. Additionally, awareness of the need to use 911 emergency telephone services increased significantly from 84% to 90% between baseline and follow-up.

At baseline, women were more likely than men to report that pain or discomfort in the jaw, neck, or back was symptomatic for AMI (72% of women, 61% of men, *P* = .02). This difference in response between men and women persisted at follow-up (94% of women, 84% of men, *P* = .006). There were no significant differences in awareness of other AMI signs and symptoms or the need to use 911 emergency telephone services by sex at baseline or follow-up (data not shown). Employees 45 years and older were more likely to recognize pain or discomfort in the jaw, neck, or back compared with younger employees at baseline (74% of older employees, 61% of younger employees, *P* = .008). Younger employees were more likely to report feeling weak, light-headed, or faint as an AMI symptom compared with older employees at baseline (87% of younger employees, 75% of older employees, *P* = .003). There were no other statistically significant differences for AMI signs and symptom awareness or the need to use 911 between younger and older employees at baseline (data not shown). At follow-up, younger employees had a higher level of awareness of the need to use 911 services compared with older employees (96% of younger employees, 87% of older employees, *P* = .006). There were no other statistically significant differences in the awareness of AMI signs and symptoms between younger and older employees at follow-up (data not shown).

**Figure 2:**
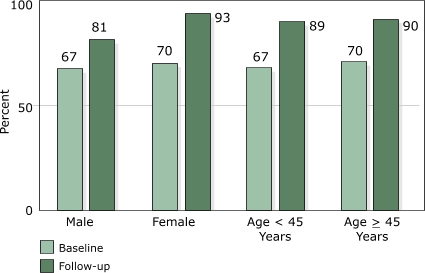
Awareness among Montana state health department employees of five or more heart attack signs and symptoms at baseline and follow-up, by sex and by age, 2003.

**Figure 3:**
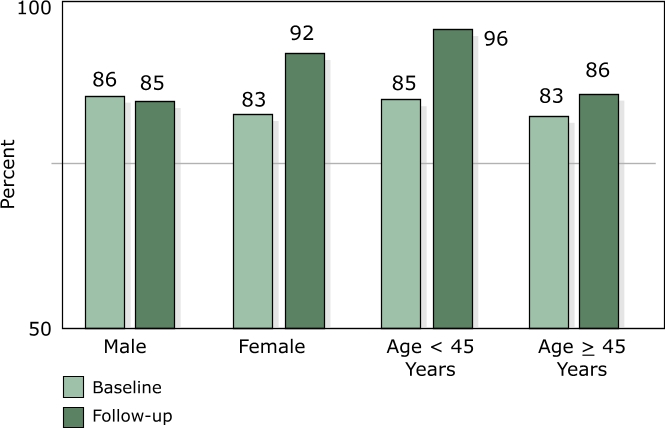
Awareness among Montana state health department employees of need to use 911 emergency telephone services if someone is having a heart attack or stroke at baseline and follow-up, by sex and by age, 2003.

The intervention was equally effective in increasing overall awareness of signs and symptoms of heart attack among men (14 percentage point increase, *P* = .02) and women (23 percentage point increase, *P* < .001) as well as younger (22 percentage point increase, *P* < .001) and older (20 percentage point increase, *P* <.001) employees from baseline to follow-up (Figure 2). Awareness of the need to use 911 emergency telephone services increased significantly in women (9 percentage point increase, *P* = .005) and younger employees (11 percentage point increase, *P* = .004), but did not change significantly in men (1 percentage point decrease, *P* = .97) or older employees (3 percentage point increase, *P* = .34) from baseline to follow-up (Figure 3).

Results of survey questions designed to assess participant awareness of intervention activities are presented in Table 3.

## Discussion

This low-cost workplace intervention was effective in increasing employee awareness of the signs and symptoms of a heart attack and the need to use 911 emergency telephone services. The intervention was equally effective in increasing awareness in both older and younger employees and had a slightly greater impact on women than men. Interestingly, the effect on increasing awareness of the need to use 911 services was found only in women and younger employees and not in men or older employees.

We were unable to identify other similar workplace intervention studies for comparison. At baseline, state health department employees were slightly more aware of signs and symptoms of heart attack and the need to use 911 compared to Montana adults overall. In a 2001 survey of Montana adults, only 60% were aware that pain or discomfort in the jaw, neck, or back were signs and symptoms of a heart attack, and 74% were aware that feeling weak, light-headed, or faint were signs and symptoms of a heart attack (17). More than 90% of adult Montanans in 2001 were aware of the signs and symptoms of chest pain, pain or discomfort in the arms or shoulders, and shortness of breath, and 82% knew to call 911 if someone is having a heart attack or stroke.

Large community intervention studies using mass media campaigns have had mixed effects on heart attack signs and symptoms awareness, use of emergency medical services, and reduction in patient delay in receiving services for persons experiencing AMI (14,18-24). A recent review of the literature provides a number of strategies for improving future community-based efforts to reduce patient delay times. These strategies include targeting high-risk groups; addressing emotional (e.g., denial) and social (e.g., inclusion of family members in education programs) issues; emphasizing cognitive aspects such as the physiologic consequences of delay; educating individuals on how to evaluate their symptoms; and developing messages specific to men and women (25). Integrating workplace awareness campaigns within larger community-based efforts may be an effective approach for reaching family and friends of persons at high risk for AMI. State health departments are attractive workplaces to pilot such interventions.

This study, however, has a number of limitations. First, all MT DPHHS employees in the three sites were exposed to the intervention, and a comparison group not receiving the intervention was not used. Other factors may have increased employee awareness outside of the intervention, although we believe that this is unlikely. Second, we used telephone surveys of employees to evaluate this intervention, and respondents were asked "aided" questions to indicate which of the possible symptoms described by the interviewer were symptoms of a heart attack. Previous studies using unaided, open-ended questions have found lower levels of heart attack awareness (26). Aided questions may overestimate awareness of signs and symptoms, and unaided questions may underestimate awareness. Third, the baseline telephone survey itself may have increased respondent awareness of the signs and symptoms. Fourth, the follow-up telephone survey took place during the summer months (July and August) and resulted in a smaller sample size (n = 337). The lack of response was due mostly to contact with answering machines, no answers, or no eligible respondent at telephone number (15%). Finally, we were not able to quantify the relative contributions of each of the intervention activities to increases in awareness.

Our findings show that this low-cost intervention can be easily replicated in other workplaces. The State of Montana will promote this type of intervention at work sites through the newly convened Governor's Council on Worklife Wellness. Increased awareness of the signs and symptoms of heart attack and the need to use 911 are important for individuals at high risk of AMI as well as family members and friends who are often the first people to have contact with persons potentially experiencing AMI.

## Figures and Tables

**Table 1:** Behavioral Risk Factor Surveillance System (BRFSS) Survey Questions on Signs and Symptoms of Heart Attack and Need to Use 911 Emergency Medical Telephone Services, Montana, 2003

**BRFSS Survey Questions^a^**	**Response Categories (*Correct Response*)**
Do you think pain or discomfort in the jaw, neck, or back are symptoms of a heart attack?	***Yes***, no, do not know/not sure, refuse to answer
Do you think feeling weak, lightheaded, or faint are symptoms of a heart attack?	
What about chest pain or discomfort...do you think these are symptoms of a heart attack?	
Pain or discomfort in the arms or shoulder (are these symptoms of a heart attack)?	
What about shortness of breath... is that a symptom of a heart attack?	
Sudden trouble seeing in one or both eyes…is this a symptom of a heart attack?	Yes, ***no***, do not know/not sure, refuse to answer
If you thought someone was having a heart attack or stroke, what is the first thing you would do? Would you:	Take them to the hospital, tell them to call their doctor, ***call 911***, call their spouse or a family member, do something else, don't know/not sure, refused to answer

a Centers for Disease Control and Prevention (16).

**Table 2:** Numbers and Percentages of Montana State Health Department Employees Reporting Awareness of Signs and Symptoms of Heart Attack at Baseline and Follow-up, 2003

	**Baseline** **n = 401**		**Follow-up** **n = 337**	

**Signs and symptoms of heart attack**	**%**	**n**	**%**	**n**

Pain or discomfort in jaw, neck, or back	69	277	91^a^	307^a^
Feeling weak, light-headed, or faint	79	318	89^a^	301^a^
Chest pain or discomfort	100	399	100	337
Sudden trouble seeing in one or both eyes^b^	43	173	46	156
Pain or discomfort in arms or shoulder	100	399	98	330
Shortness of breath	92	370	95	319
Five or more correct responses to heart attack signs and symptoms^c^	69	277	89^a^	301^a^
Call 911 if someone is having a heart attack or stroke	84	337	90^a^	303^a^

a *P* ≤ .05.

b Correct response is "no."

c Total of six questions.

**Table 3:** Results of Survey Questions to State Health Department Employees, Montana, 2003

**Survey Question**	**% Responding Yes**
In the past two months, have you seen posters in your workplace with information on heart attack signs and symptoms?	98
**Those who responded yes to question above were asked, "Where did you see these posters?"**	
Hallways	67
Stairwells	16
Elevators	15
Bathrooms	96
Did you participate in any of the weekly e-mail quiz contests on heart attack signs and symptoms?	63
In the past two months, did you receive an *Act in Time* brochure and wallet card on heart attack signs and symptoms with your paycheck?	74
